# Supplementary material for: Validating Antibodies for Quantitative Western Blot Measurements with Microwestern Array
Source: Sci Rep. 2018 Jul 27;8:11329. doi: 10.1038/s41598-018-29436-0 (PMC6063895; doi:10.1038/s41598-018-29436-0)
Supplement: Supplementary file 1 — Supplementary Information [file 41598_2018_29436_MOESM1_ESM.pdf]

Validating Antibodies for Quantitative Western Blot Measurements with Microwestern Array

Rick J. Koch, Anne Marie Barrette, Alan D. Stern, Bin Hu, Mehdi Bouhaddou, Evren U. Azeloglu,
Ravi Iyengar, Marc R. Birtwistle

The five supplementary figures correspond to full scans of the images presented throughout the main text. These are followed by the SOP's related to running a microwestern array.

Full scan of the MW Ladder blot used to measure pipetting repeatability (from Fig. 2).

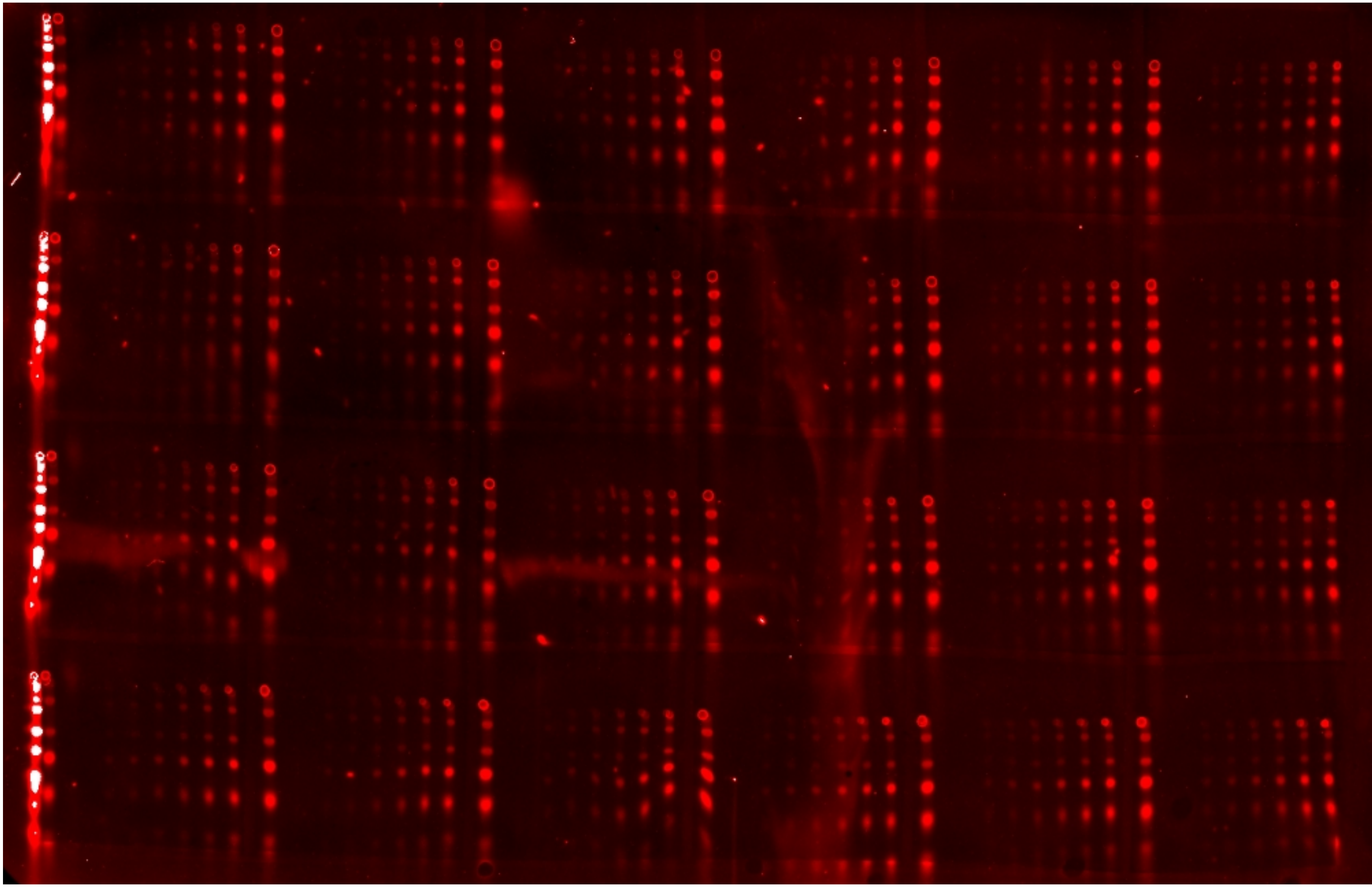

Full scan of the Ab validation using 24 well format used in text Figures 3 and 5.

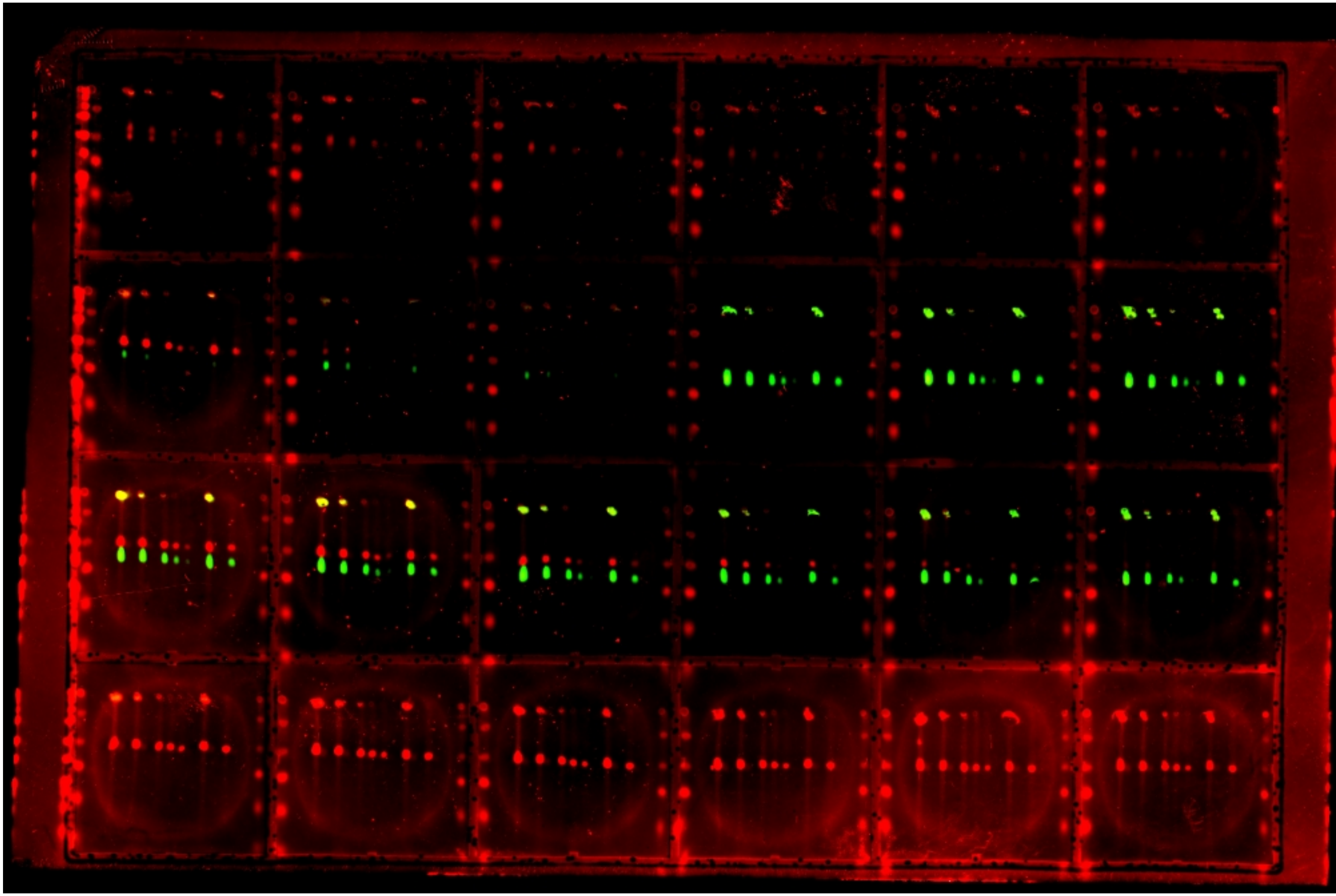

Full scan of the Ab validation using 96 well format showing wells H1 and F2 in Fig. 4.

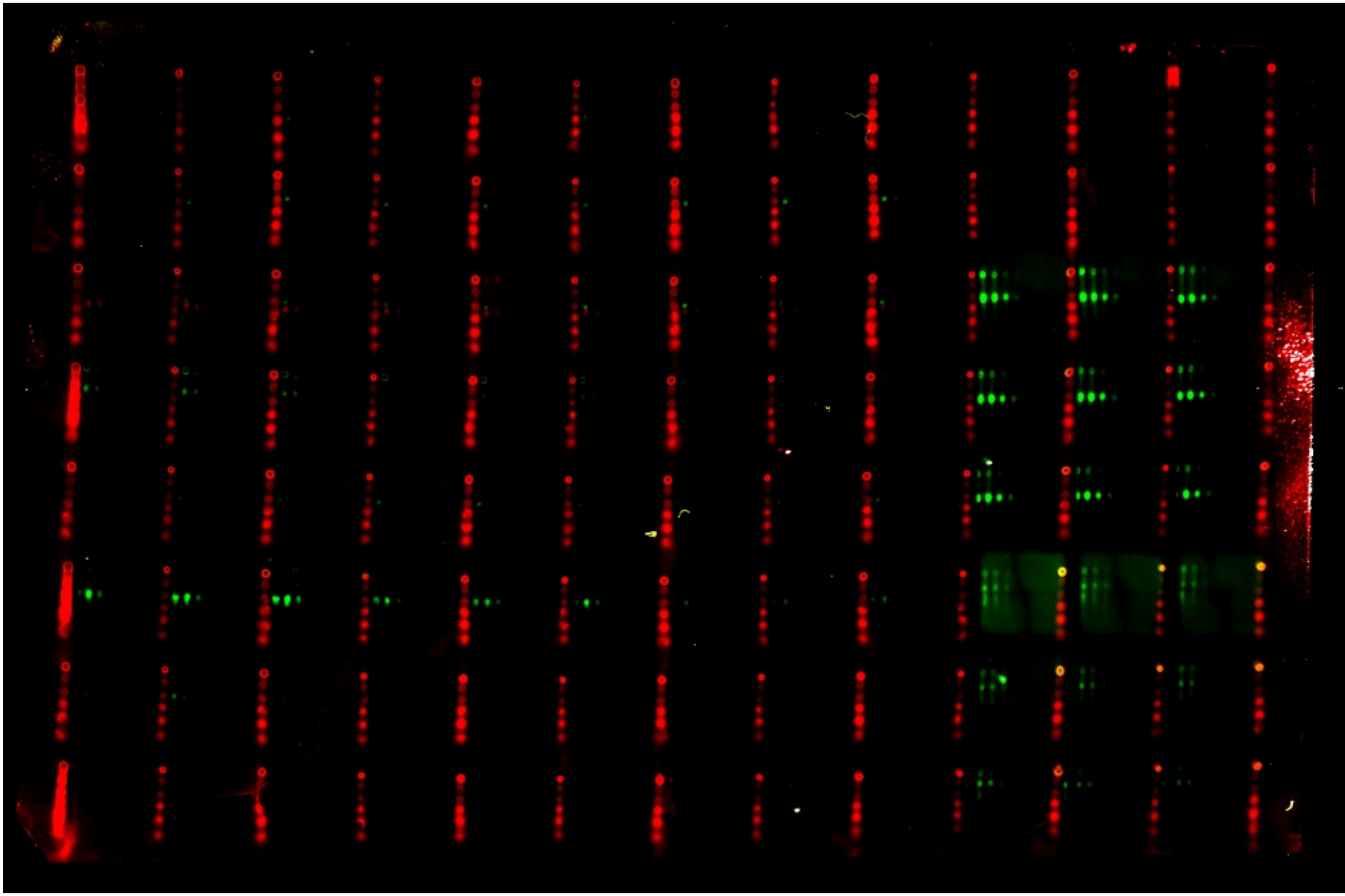

Full scan of the Ab validation using 24 well setup showing results for EGFR in Fig. 4.

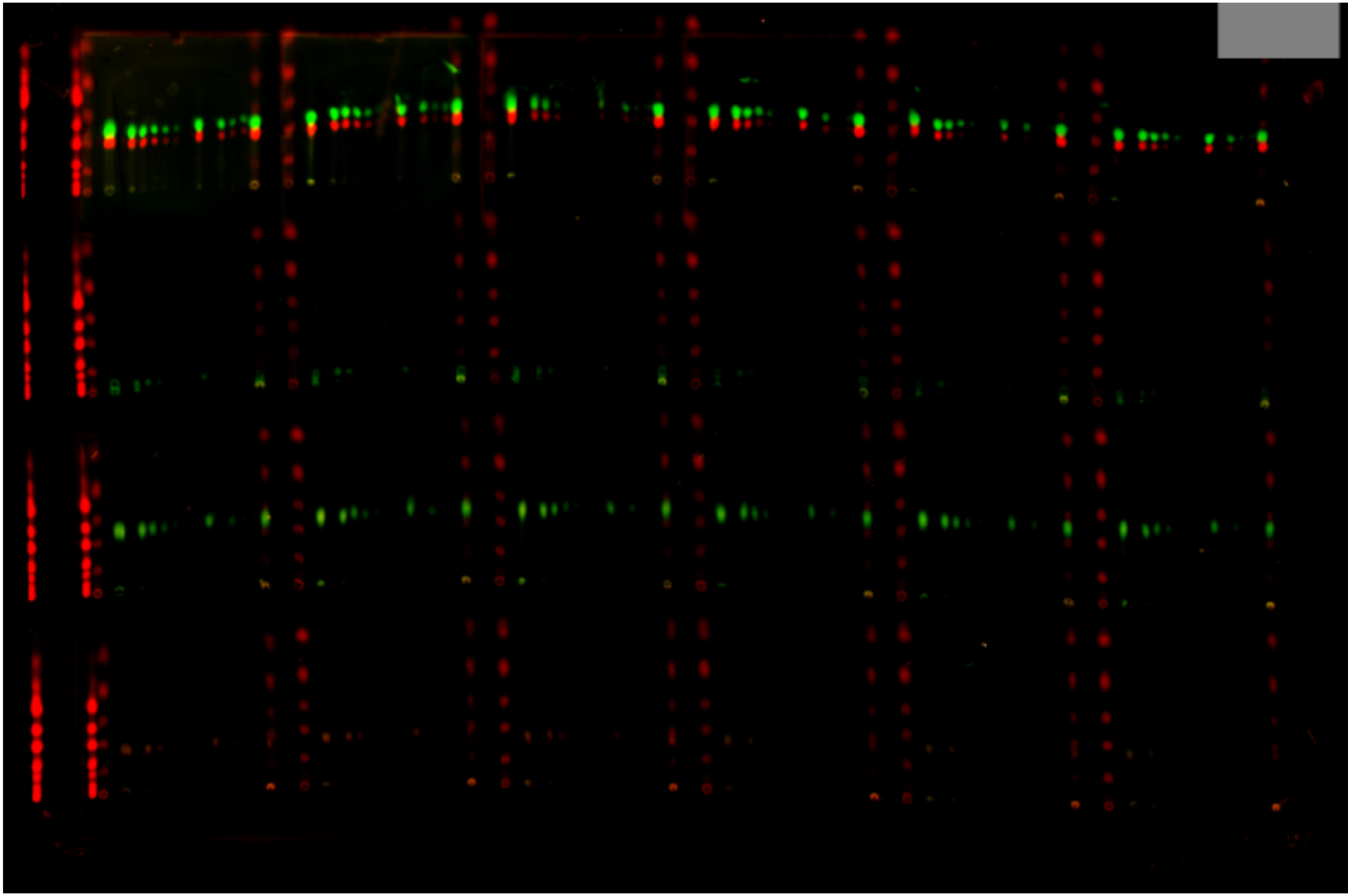

Full scan for Ab validation showing wells D1-D9 for NFAT4 in Fig. 4.

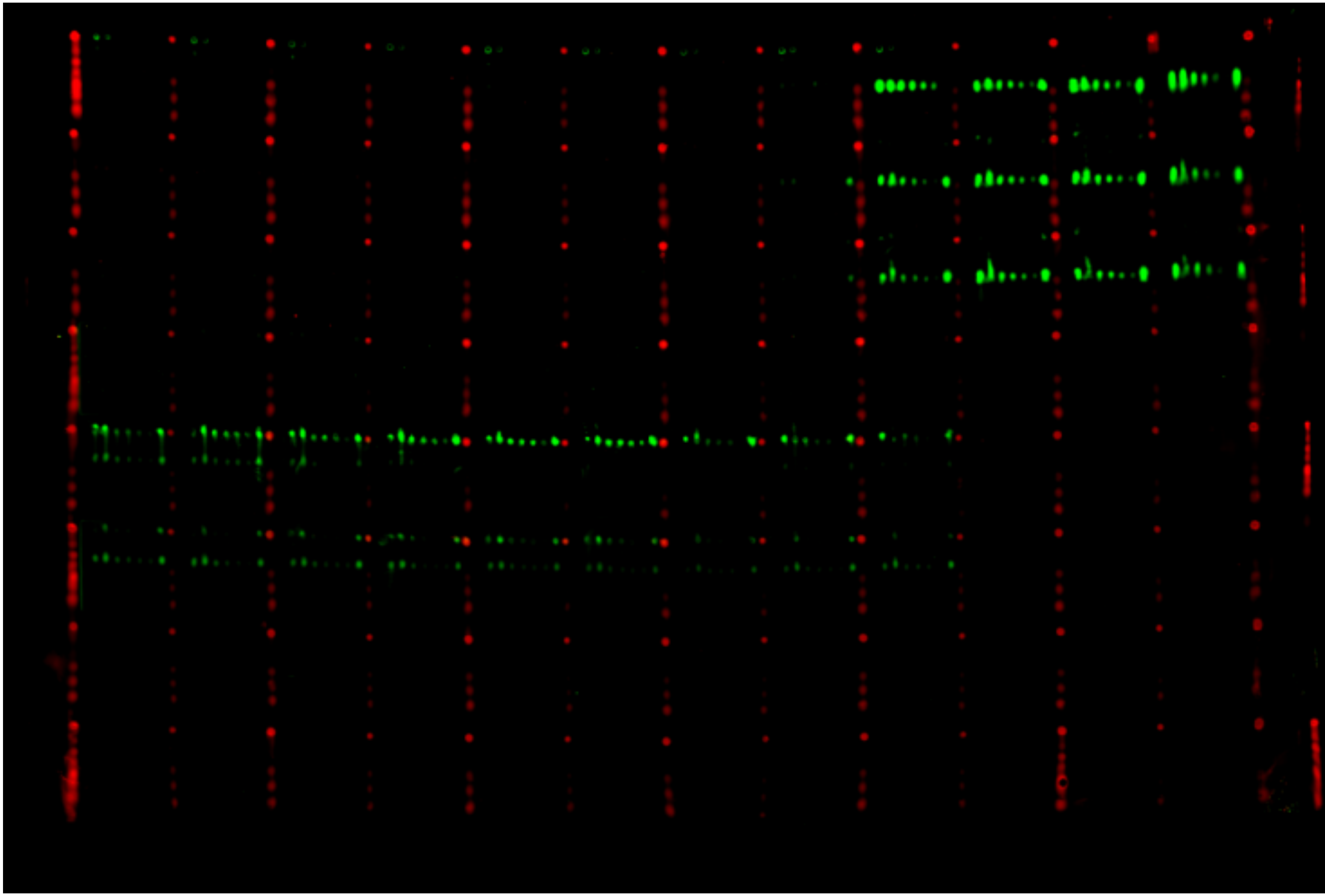

Icahn School of Medicine at Mount Sinai LINCS Center for Drug Toxicity Signatures

Standard Operating Procedure: Casting of Gels for Microwestern Array

DToxS SOP Index: A-9.0
Last Revision: 02/22/2016
Written By: Mark Ciaccio and Rick Koch

Approvals (Date): Joseph Goldfarb (pending)
Marc Birtwistle (2/22/16)
Eric Sobie (02/22/2016)
Ravi Iyengar (2/22/16)

Quality Assurance/Control (QA/QC) steps are indicated with green highlight.

Metadata recording is highlighted with yellow highlight and superscript indices.

NOTE: This protocol was inspired by that written at U. Chicago by Mark Ciaccio (Ciaccio MF, Wagner JP, Chuu CP, Lauffenburger DA, Jones RB (2010) Systems analysis of EGF receptor signaling dynamics with microwestern arrays. Nat Methods 7:148–155; Youtube videos on MWA, www.youtube.com/watch?v=0iUhoWL1IC0).

1) Set up the Gel Cast
   a) Clean long and short glass plates (Moliterno, long plate, #GBS-160-280, 16 cm x 28 cm x 2.3 mm and short plate, #GBS-160-270, 16 cm x 27 cm x 2.3 mm)
      i) Add MilliQ water (Millipore Advantage A10 system 18.2 MΩ) to one side of each plate and wipe dry with Chemwipes (Fisher, #06-666-A)
      ii) Apply 95% EtOH (Fisher, #S25309E[1]) to the same surfaces and squeegee with a single edge razor blade (Fisher, #12640) to remove any particulate matter. Remove excess EtOH with Chemwipes.
   b) Silanize each cleaned surface
      i) Prepare a 2.5% silane stock by combining 1.25 mL dichlorodimethylsilane (Sigma-Aldrich, #40140-25ml[2]) and 48.75 mL 100% ethanol (Fisher, #04-355-22) in a 50 mL conical tube (Fisher,#14-432-22). Store wrapped in aluminum foil at room temperature. Record the date of preparation on the tube. The solution is good for up to 6 months.
      ii) Apply approximately 0.5 mL of 2.5% silane to each cleaned side of the glass plates by spreading drops over the surface.
      iii) Spread silane evenly over the glass surface with Chemwipes until dry. Clean surface with MilliQ water and Chemwipes as before (Step *1ai*)

c) Place 0.4 mm deep plastic spacers (30 cm x 1.5 cm x 0.4 mm), made from white high impact styrene,on the long edges (28 cm) of the long plate
d) Center Netfix rectangle, 26.5 cm x 12.5 cm (Serva, #42500.01) between spacers.
e) Place short plate with the silanized side down on top of the spacers.
f) Tape (¾” TapeLogic vinyl electrical tape,yellow-- Staples #191530) sides and bottom of the plates and place 3 clamps (Staples #831610) along each long side (6 total) equally spaced. Even spacing ensures that the gel will have uniform thickness. (See Figure 1, below.)

Figure 1.

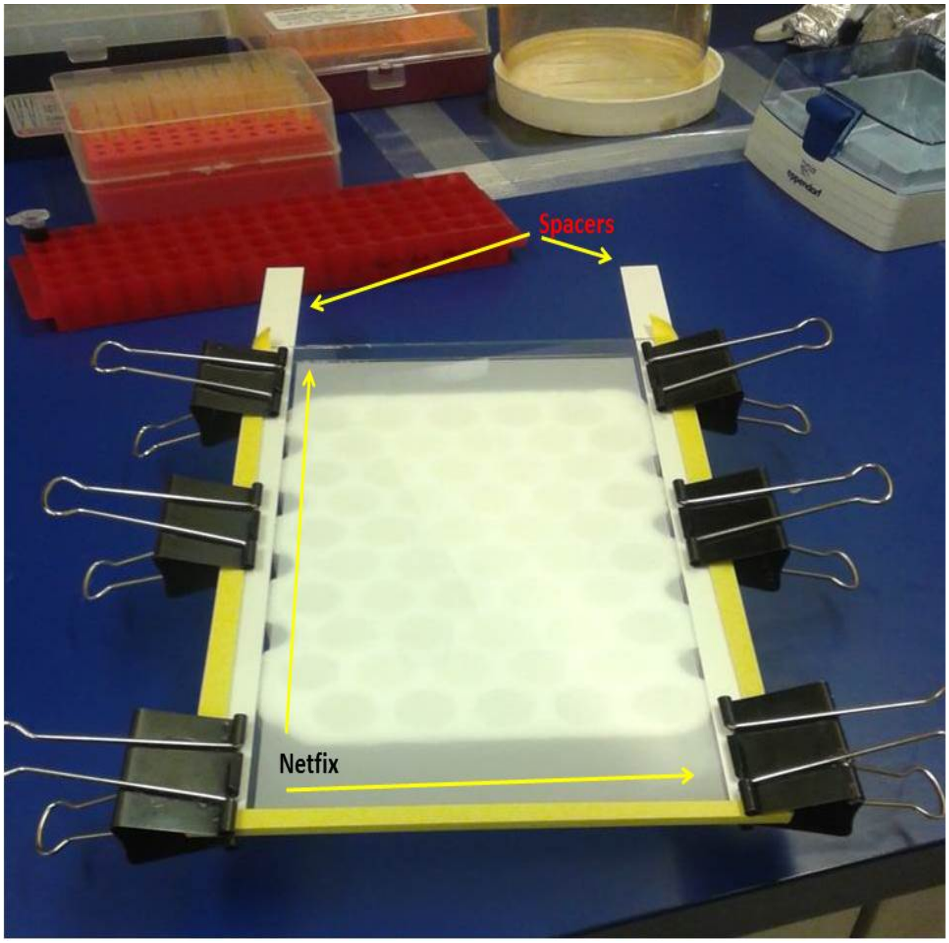

2) Cast the Gel
   a) Make 5x Gel Buffer, pH 6.9: 1.2M Tris Acetate (Tris; BioRad #161-0719[3]) (Acetic acid; Sigma-Aldrich #320099[4])
      i) Add 145.4 g Tris base (BioRad, #161-0719) to 700 mL MilliQ water. pH should be between 11.0 to 11.4. If it is not, start fresh.
      ii) Add 65 mL glacial acetic acid (AA) (Sigma-Aldrich, #320099) while monitoring pH; pH should go down to ~7.1.
      iii) Let solution stand overnight.
      iv) Add AA in 0.5 mL increments until pH reaches 6.9.
      v) Let solution stand for at least one hour at room temperature.
      vi) Repeat steps iv) and v) until pH is stable at 6.9.
      vii) Bring volume up to 1 L with MilliQ water and store at 4°C. This stock will be good for 6 months.
      viii) Do not add sodium hydroxide (NaOH) to adjust pH. The ions may affect protein migration during electrophoresis. If the pH stably becomes <6.75 then restart at Step *2ai* and add smaller increments of AA in Step *2aiv*.
   b) In a 50 mL conical tube vigorously mix the following reagents to produce a 9.5% acrylamide gel: 9.5 mL acrylamide/bis solution (BioRad, #161-0156[5]; 30% Acrylamide/Bis Solution, 29:1); 8 mL MilliQ water; 6 mL glycerol (Sigma #G5516-500ml) and 6 mL 5x Gel Buffer.
   c) Allow bubbles to subside by letting solution stand uncovered at room temperature for about 10 minutes. Add 0.3 mL 10% SDS (Fisher #BP 2436[6]); 0.15 mL 10% Ammonium Persulfate (APS) and 0.012 mL TEMED (BioRad #161-0800[7])
      i) APS (BioRad #161-0700[8]) 10% is made as follows: dissolve 1 g ammonium persulfate (FW 228.2) in a final volume of 10 mL MilliQ water. Store covered at 4˚C (make fresh weekly).
3) Mix gently but thoroughly by inverting, and then pour into the cast from above. The cast should be held at about a 45° angle with the bench top surface. Keep excess gel solution in the 50 mL tube at room temperature to verify polymerization. Let stand 1 hour at room temperature.
4) Cutting the Gel
   a) Carefully remove clamps and tape from around the cast.
   b) Remove the top plate carefully with a gel wedge or razor blade.
   c) Remove gel from the bottom glass plate by lifting manually from the plate with gloves.
   d) Place the gel between 2 plastic sheets (3M Transparency Film, 8.5" x 11", #AF4300). Press out bubbles between the plastic and the gel using a rubber brayer (Staples, #WYF078276230510), pressing firmly yet without excess pressure. Excess is defined as that which causes the gel to deform.
   e) Use a paper cutter (Staples, #103450) suitable to cut plastic to remove the Netfix border.
   f) Divide the remaining gel into two equal sections (each must be at least 11.5 cm wide for a 96 well hybridization plate; bigger is better).
   g) Store each section in an individual plastic resealable bag, removing air by hand.
   h) Record the date on the bag. Gels are stable for 3months at 4°C.

Metadata

Record lot # for the following:

The specific lot #'s used in our experiments (as of July 2015) are:

1. 95% EtOH (Fisher, #S25309E) Lot #4AJC14I23001

2. Dichlorodimethylsilane (Sigma-Aldrich, #40140-25ml) Lot #BCBH8051V

3.Tris (BioRad #161-0719) Control #210003844

4. Acetic acid (Sigma-Aldrich #320099) Lot #SHBC4388V

5. Acrylamide/Bis Solution, 29:1 (BioRad#161-0156; 30%) Control #200007829

6. 10% SDS (Fisher #BP 2436) Lot #121099

7. TEMED (BioRad #161-0800) Control #210008697

8. APS (BioRad #161-0700)Control #210009085

Icahn School of Medicine at Mount Sinai LINCS Center for Drug Toxicity Signatures

Standard Operating Procedure:
Preparation of Adherent Cell Lysates for Microwestern Array

DToxS SOP Index: A-10.0
Last Revision: 09/15/2016
Written By: Marc Ciaccio (U. Chicago) and Rick Koch

Approvals (Date): Joseph Goldfarb (DATE)
Marc Birtwistle (9/14/2016)
Eric Sobie (DATE)
Ravi Iyengar (DATE)

Quality Assurance/Control (QA/QC) steps are indicated with green highlight.

Metadata recording is highlighted with yellow highlight and superscript indices.

NOTE: This protocol was inspired by that written by Mark Ciaccio (Ciaccio MF, Wagner JP, Chuu CP, Lauffenburger DA, Jones RB (2010) Systems analysis of EGF receptor signaling dynamics with microwestern arrays. Nat Methods 7:148–155; Youtube videos on MWA, www.youtube.com/watch?v=0iUhoWL1IC0).

1) Prepare inhibitor stock solutions and reagents. Aliquots are single use for a 10 mL batch of lysis buffer and can be scaled accordingly. For optimal function do not freeze-thaw aliquots but rather safely discard any unused portion.
   a) 200 mM activated sodium orthovanadate (Na_3VO_4)(FIVEphoton Biochemicals #ActVO-[1]):
      i) Aliquot the activated sodium orthovanadate (60 per aliquot) and store the aliquots at -20°C. Good for ~1 year (per manufacturer).
      ii) QA/QC 1. Check color of final solution. It must be colorless. If not, return to the company.
   b) 1mg/mL Aprotinin
      i) Weigh 1mg Aprotinin (Fisher/MP Biomedicals #194559[2]) into a 1.7mL microcentrifuge tube (VWR, #20172-698).
      ii) Add MilliQ water (Millipore Advantage A10 system 18.2 MΩ) to bring final volume to 1mL.
      iii) Aliquot for single use (12 μL per aliquot into a 0.6mL microcentrifuge tube (Fisher #05-408-120). Store at -20°C for up to one year.
   c) 1mg/mL Leupeptin
      i) Weigh 1mg leupeptin (MP Biochemicals, #195624[3]) into a 1.7mL microcentrifuge tube.
      ii) Add MilliQ water to bring final volume to 1mL.

iii) Aliquot for single use (12 μL per aliquot into a 0.6mL microcentrifuge tube). Store at -20°C for up to one year.

d) 1mg/mL Pepstatin A
   i) Weigh 1mg pepstatin A (MP Biochemicals, #195368[4]) into a 1.7mL microcentrifuge tube
   ii) Add MilliQ water to bring final volume to 1mL.
   iii) Aliquot for single use (12 μL per aliquot into a 0.6mL microcentrifuge tube). Store at -20°C for up to one year.
e) 1M ß-glycerophosphate (ß-GP)
   i) Weigh 1.53g ß-GP (Santa Cruz Biotechnology #sc203323[5]) into a 15 mL conical tube (Fisher, #50-754-1410)
   ii) Add MilliQ water to bring final volume to 5mL.
   iii) Aliquot for single use (110 μL per aliquot into a 0.6mL microcentrifuge tube). Store at -20°C for up to one year.
f) 100mM Ethylenediaminetetraacetic acid (EDTA)
   i) Weigh 146 mg EDTA (Sigma # E6758[6]) into a 15 mL conical tube
   ii) Add MilliQ water to about 3 mL.
   iii) Adjust pH to 8.0 with 10 M NaOH (Fisher #SS255-1[7])
   iv) Bring final volume to 5 mL with MilliQ water.
   v) Aliquot for single use (110 μL per aliquot into a 0.6 mL microfuge tube). Store at -20°C for up to one year.

2) Prepare 10 mL of lysis buffer.
   a) Note: Lysis buffer should be prepared fresh the day of the cell lysis. Recipe can be scaled accordingly.
   b) Combine 2 mL of stock 5x Gel Buffer (SOP A-9.0, step 2), 1 mL of 10% Sodium Dodecyl Sulfate (SDS) (Fisher #BP2436-1[8]), 50 μL glycerol (Sigma, #G5516[9]), 100 μL of stock 100 mM EDTA (step 1f) and 7 mL of MilliQ water in a 15 mL conical centrifuge tube.
   c) Put tube on ice and allow it to cool.
3) Cell lysis
   a) NOTE: For a 24 well downstream microwestern experiment, we find that ~ 150 μL at 1 mg/mL total protein concentration is needed after lysis (or equivalently e.g. 300 μL at 0.5 mg/mL). One should empirically determine the number of cells to plate prior to preparation for microwestern by measuring protein concentration as in Step 4g below. We recommend 500k cells as a conservative starting point but routinely see adequate results with as low as 150k cells. Optimization to reduce these amounts is ongoing, and of course everything depends on the abundance of the epitopes and antibody quality.
   b) Prepare cells for adding the lysis buffer. This typically consists of washing cells 2x in ice-cold phosphate-buffered saline (PBS) (Corning #21-040-CV) [11].
      i) Place plate / dishes on ice.
      ii) Add 5 mL ice-cold PBS per 10 cm dish (scale volume according to culture area for different size dishes). Aspirate fully. Repeat. It is important at this stage that aspiration is complete.
   c) Add 1.0 mL ice-cold lysis buffer per 10 cm dish (scale volume by culture area for different size dishes).

d) Scrape cells with a cell scraper (Denville Scientific, #T0139), comprehensively going over the entire culture area multiple times. Tilt the dish / plate by leaning it on the side of the ice tray at an approximately 30 degree angle and allow lysate to gravity pool for at least 20 to 30 seconds (can leave up to ~10 minutes if lysing batches of dishes).
e) Transfer lysate into a pre-cooled 1.7 mL microcentrifuge tube using a pipette. Keep on ice.
    i) NOTE: At this point, the lysate at the end of step d) may be applied to a separate dish which has gone through step b), to increase the protein concentration.
    ii) Pipette carefully to avoid introducing bubbles into the lysate, which increases difficulty of maximum collection.
f) Place lysate-containing tube into a 95°C heat block (Fisher, #11-718-20) for 5 minutes. Vortex for ~10 sec. At this point, one can freeze sample at -80°C if necessary.

4) Process and concentrate lysate
    a) If lysates are frozen, thaw on ice for at least 10 min. Vortex briefly (~two seconds)..
    b) Sonicate. There are two options for sonication, depending on the lysate volume.
        i) Large volumes (>~ 1 mL). Sonicate at Power 9 on a probe sonicator (Misonix; model XL2000) for one second while keeping lysate on ice. Turn off sonicator for 1 sec. Repeat on / off cycle 9 more times (20 seconds total).
        ii) Small volumes use the VialTweeter from Hielscher Ultrasonics placed in a 4^{0}C cold room. Note the sonotrode is machined by the company for specific microfuge tubes. The tubes we use are listed below.
            (1) Place lysates up to 200 μL in 0.25 mL polypropylene tubes (Fisher #02-681-230).
            (2) Place tubes into sonotrode and sonicate for 10 seconds followed by a 30 second rest period. Repeat this cycle 10x. Amplitude is set at 100%.
            (3) For lysates with a volume of 200 μL to 500 μL we recommend a Protein LoBind 1.5mL tube (Eppendorf #022431081); however, note we have not yet optimized the VialTweeter for these tubes and volumes.
        iii) NOTE: The microwestern array facility at Univ. of Chicago uses a Covaris bath sonication apparatus.
    c) Large volumes (step 4.b.i above) are needle sheered with a 25 gauge needle (Fisher, #NC0779671), by drawing the sample into and out of the needle and a 1.0 mL plastic syringe five times (Fisher #22-253-260).
    d) Place tubes into a 95°C heat block for 2 minutes and immediately place in 500 μL Centricon spin columns (Amicon Ultra – 0.5mL centrifugal filter; Millipore #UFC501096).
    e) Spin at 14,000g, room temperature for 15 min.
    f) Each sample should be concentrated ~5X to 10X by volume. If not, spin longer.
    g) As per the protocol for the Amicon centrifugal filters, recover concentrated samples by placing the filter device upside down in one clean microcentrifuge tube (tube included in the Amicon kit) and spin at 1,000g for 2 minutes.
    h) Freeze at -80°C or proceed directly to measuring protein concentration using the Pierce 660nm Protein Assay kit, (Fisher #22660[12] and Fisher #22663[13]). Follow the manufacturer's protocol. We perform triplicates.

i) QA/QC 2. Check the turbidity of the lysate after prepared for Pierce assay. If it is turbid or "clumpy" this suggests inadequate sonication and/or not enough lysis buffer volume was used during cell lysis.

ii) QA/QC 3. Check total protein concentration. We aim for 5mg/mL of total protein, but have had suitable blotting results with as little as 1 mg/mL. If protein concentration is too low, repeat steps d) – f).

i) Equalize protein concentration of all lysates with ice-cold lysis buffer. Aliquot lysates into 1.7 mL microfuge tubes, if necessary, to avoid repeated freeze-thaw cycles (21 μL aliquots work well). Freeze at -80°C until use.

Metadata

Record lot # for the following:

The specific lot #'s used in our experiments (as of July 2015) are:

1. Sodium Orthovanadate (FIVEphoton Biochemicals) Lot #26711-1
2. Aprotinin (Fisher/MP Biomedicals #194559) Lot #MR30363
3. Leupeptin (Fisher/MP Biochemicals, #195624) Lot #M8546
4. Pepstatin A (Fisher/MP Biochemicals, #195368) Lot #8372K
5. ß- GP (Santa Cruz Biotechnology #sc203323) Lot #H1115
6. EDTA (Sigma # E6758) Lot # 011M0123V
7. NaOH 10N (Fisher #SS255-1) Lot # 932721-247.
8. 10% SDS (Fisher #BP2436-1) Lot #121099
9. Glycerol (Sigma, #G5516) Lot #SHBC7796V
10. 1M DTT (Fisher, #AC426380100). Lot #A0345819
11. PBS (Corning #21-040-CV) Lot # 12815001
12. Pierce 660nm Protein Assay Reagent (Thermo Scientific # 22660) Lot # QE216003
13. Ionic Detergent Compatibility reagent (Fisher #22663). Lot #QG220132

Quality Assurance/ Quality Control

QA/QC 1. Yellow tinge indicates Na Orthovanadate is not activated.

QA/QC 2. We have found that if one can see “clumps” or if the solution is turbid immediately before absorbance measurements in the Pierce 660 assay, then protein concentration measurements will be unreliable as also might be the microwestern results.

QA/QC 3. Be mindful that a minimum volume for adequate nanoplotter dispensing performance is ~18 uL in the microtiter plate. We pipette 18 uL into the plate from 21 uL in the tube. Higher protein concentration gives a better signal but sample must have low viscosity for the arraying process to be functional. All of this also depends on the cell type and antibodies, so there is often some optimization and iteration.

Icahn School of Medicine at Mount Sinai LINCS Center for Drug Toxicity Signatures

Standard Operating Procedure:
Printing of Samples for Microwestern Array

DToxS SOP Index: A -11.0
Last Revision: 11/10/2016
Written By: Mark Ciaccio and Rick Koch
Approvals (Date): Joseph Goldfarb (DATE)
Marc Birtwistle (11/10/2016)
Eric Sobie (DATE)
Ravi Iyengar (DATE)

Quality Assurance/Control (QA/QC) steps are indicated with green highlight.

Metadata recording is highlighted with yellow highlight and superscript indices.

Pre-requisite Note: We use the GeSim Nanoplotter Model 2.1E; GeSim software NPC16 V2.15.53 and Short Manual for Version 2.15.50 and later.

Printing requires the choice of an appropriate work plate, transfer file, and pipetting program depending on the experiment. A work plate graphically defines all the objects (microtiter plate(s), gel(s), wash station, stroboscope) in x, y space on the Nanoplotter platform (See p.65, Short Manual). We use the work plate, “050415RK_24 Well.npw” when using the 24 well hybridization plate. A transfer file specifies which samples are spotted in a particular position on the gel (See p. 94 and p. 129, Short Manual). Transfer files generally vary, reflecting the number of samples to be printed and the positioning of those samples on the gel. The pipetting program determines how the pipette(s) pick up the sample(s) and spot the gel (See p. 129, Short Manual, we use “TransferSimMultiPlates_074” program). Our files are freely available upon request, and appropriate choices are described in the SOP. Creating new files is beyond the scope of our SOPs.

1) Load the appropriate work plate file and ensure the transfer file is properly set up.
    a) Click “Work Plate,” open Work Plate settings and, in our set up, in “RK workplate files” folder choose “050415RK_24 Well.npw” and click “Load.”
    b) Run the transfer file in Simulation mode (section 4.5, GeSim short manual). We recommend doing this the first time a transfer file is used.
    c) Our transfer files are set up to deposit 15 drops per spot, and 10 cycles of spots. We draw 1.5 uL of lysate into the tip every cycle, and discard the unused lysate (most of it). There is a recycle option available but so far we have not optimized it.

2) Make 1.0 L 5X Gel Buffer (SOP#A-9.0—step 2a), if not already made up.
    a) Note: 5X Gel Buffer must stand overnight so prepare this the day prior.

3) Turn on humidifier (Airwin UB1 CT) to 75%.

4) Prepare 100 mL of Rehydration buffer.
   a) In a 125 mL Erlenmeyer flask add 57 mL MilliQ water (Millipore Advantage A10 system 18.2 MΩ); 20 mL of glycerol (Sigma #G5516-500ml[1]) and 20 mL of 5X Gel buffer. Shake to completely mix glycerol.
   b) Add 1 mL 10% SDS (Fisher #BP 2436[2]); 1 mL 1 M sodium bisulfate (Fluka #71656[3]) and 1 mL 1M DTT (Fisher, #AC42638-100[4]).

5) Place the 100 mL of Rehydration buffer into a clean glass tray (Fisher #15-242B). Take a gel from 4^{0}C storage (see SOP # A-9.0), remove one of the plastic sheets, and rehydrate with uncovered gel side facing up for 5 minutes in rehydration buffer in glass tray. The gel should be completely immersed.

6) Take gel out of tray and blot off excess buffer by touching gel edge to paper towels at an angle. Save buffer for step 2d in SOP A-13.0 (Gel Electrophoresis).

7) Place the gel with uncovered side up onto the Nanoplotter platform as shown in Figure 1 below. Use the Positioning slide to orient gel in the correct x,y coordinates (refer to Work plate for the positioning for your setup).

Fig. 1: Illustration of Gel Placed on GeSim Platform prior to Printing

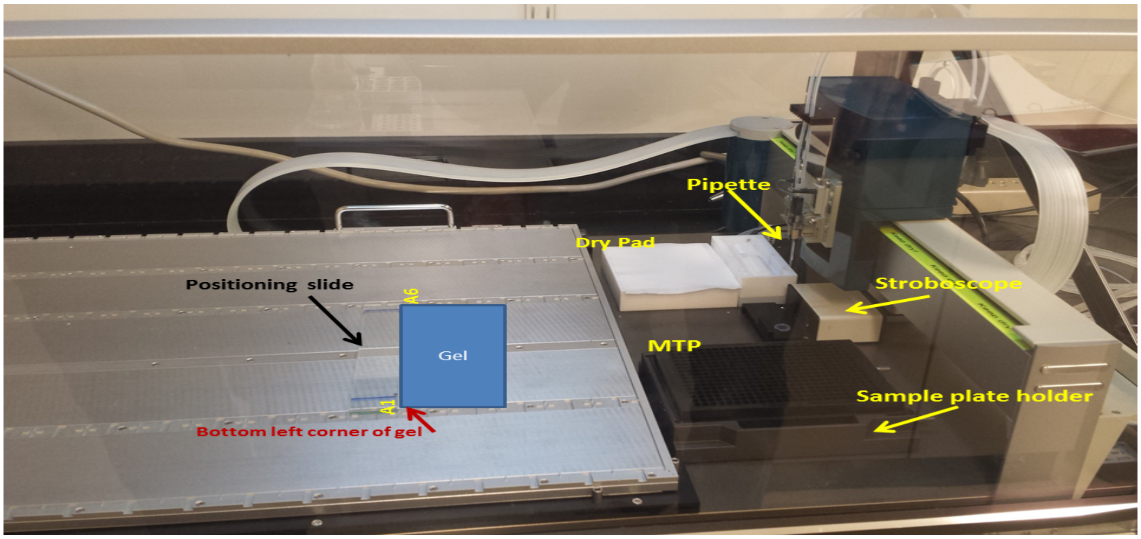

8) Gently roll out bubbles between gel and bottom plastic sheet using roller from BioRad Criterion blotter kit (BioRad#1704070), and allow gel to sit in the humidified chamber for 10 minutes before printing.
   a) Make sure there are no visible puddles of fluid on the gel. If so, repeat Steps 6-8.

9) Take nanoplotter out of "Standby" mode by first clicking "STOP", and then clicking "R" (see Fig 2, below).

Fig 2: Screenshot of Standby Mode

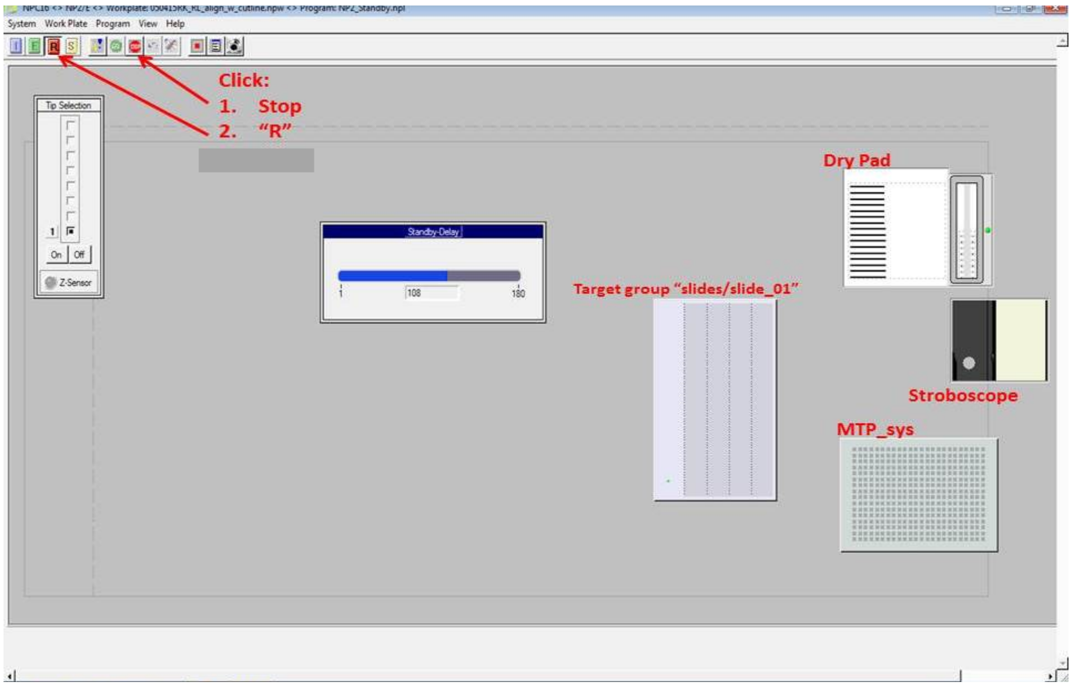

10) QA/QC1: Check drop alignment.

a) Click “I” mode (see Fig 3 below). This permits water to be ejected from the nanoplotter tip when selected in step c.

b) Click “Stroboscope” icon opening “Stroboscope” window (see Fig 3 below)

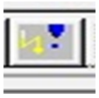

c) Click on “Tip 1” box in stroboscope window (see Fig 3) to open camera view window and bring pipette into view.

d) If droplets are aligned as shown below in Fig 3 “Camera View” window, click “Close” in Stroboscope window. (The 3 droplets shown are a “drop”, defined as what is ejected from the nanoplotter pipette due to a single pulse.)

Fig. 3: Screenshot After Opening Stroboscope and Camera View Windows

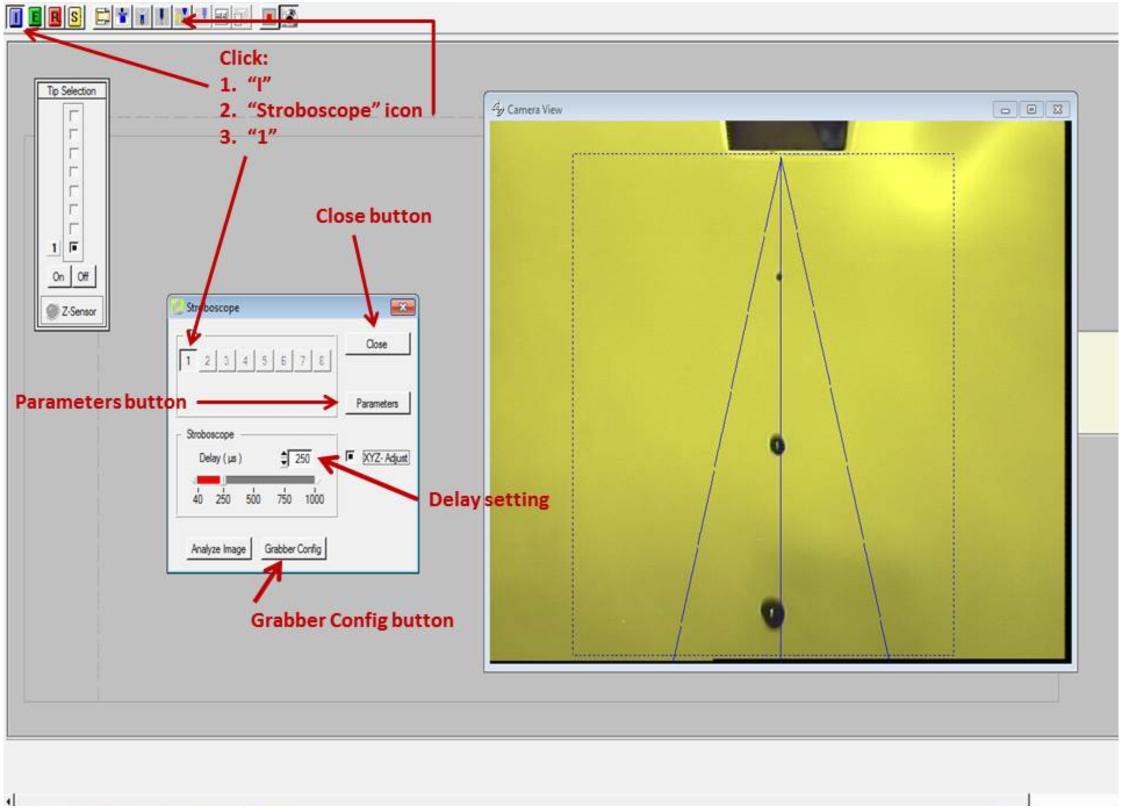

e) If droplet is not aligned this may be due to improper tip position for spotting the gel. The following steps correct the alignment.

i) Slide "Delay" (see Fig. 3) to 40 µs (from default of 250 µs) to see droplet ejected from the nozzle of the pipette tip as shown below in Fig.4.

ii) Click "Grabber Config" tab shown in Fig. 3 above which produces the "Grabber Config screen shot", below, Fig. 4.

iii) In new window press "Tip Position." The cursor changes to a crosshair.

iv) Click on the spot just below the nozzle position.

v) Close the window. When new popup screen asks to "save the new setting" click ok.

vi) In Stroboscope window (see Fig. 3), click on "Parameters" and click "Set Defaults." Clicking "Set Defaults" will reset the "Delay" setting back to 250 µs. If droplet is now aligned properly close Stroboscope window.

Fig. 4: Grabber Config screen shot

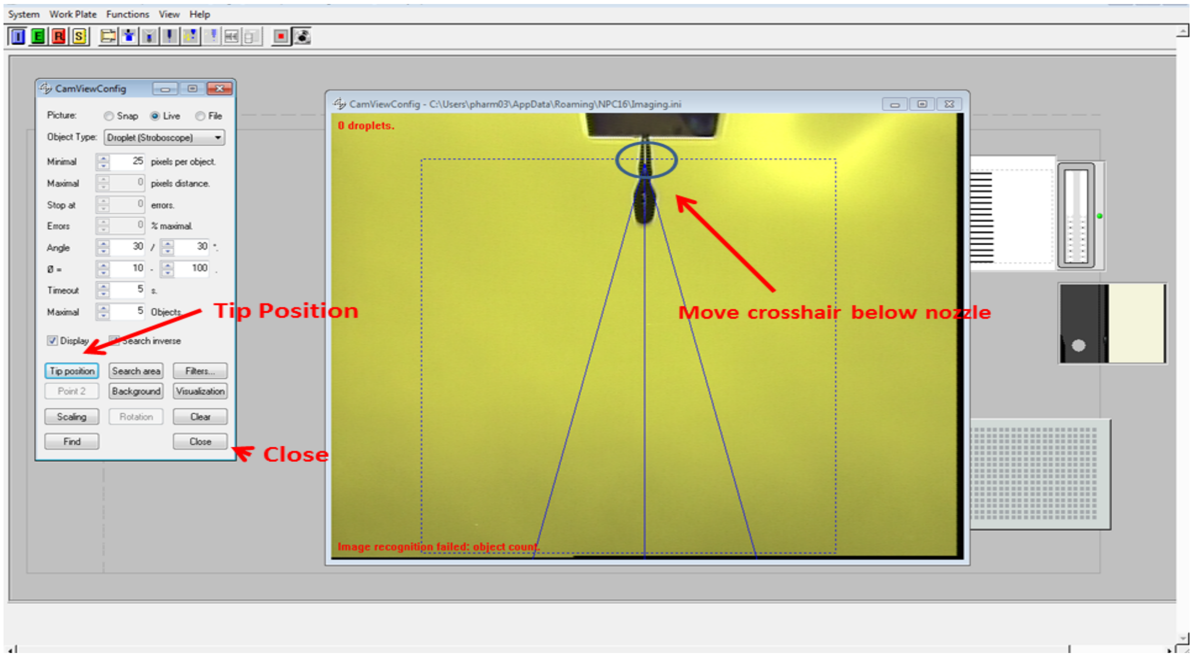

11) Open the desired Work plate. Click “Work Plate” on tool bar (top row of Fig. 4 to the left). Click “Open Work Plate Settings …” and choose the appropriate work plate file from the folder. The nanoplotter may already have the file loaded. The work plate chosen depends on the eventual hybridization plate to be used. We use “091715RK_Target_24well.npw” for a 24 well plate.

12) Before printing, allow sample lysates to thaw on ice. Mix 12 µL LI-COR protein molecular weight ladder (LiCor #928-70000[5]), 1 to 1 with lysis buffer without inhibitors (see SOP A-10.0 Preparation of Adherent Cell Lysates Step 2a). Heat lysates at 95°C in a dry heat block for 2 minutes. Centrifuge at 14,000g for 2 minutes to collect condensation and settle debris (if present).

13) Place 24 µL of LI-COR protein ladder in lysis buffer in A1 of the 384-well black microtiter plate (Corning #4514). Load subsequent samples horizontally in the A2, A3, A4, etc. plate locations, 24 µl per well, according to the transfer file format being used. Load 24 µl of Bromophenol blue (0.1% in water) in a well after the samples.

14) Place the 384-well plate flat into the sample plate holder (see Fig. 1 above) in the GeSiM arrayer.

15) Click “R”, click “Run Npl application” icon and choose “TransferSimMultiPlates_07A4” in “Run Application/Function” window. (Fig 5, below.) This run program is used for all plate configurations.

Fig. 5 : “Run Application/Function:

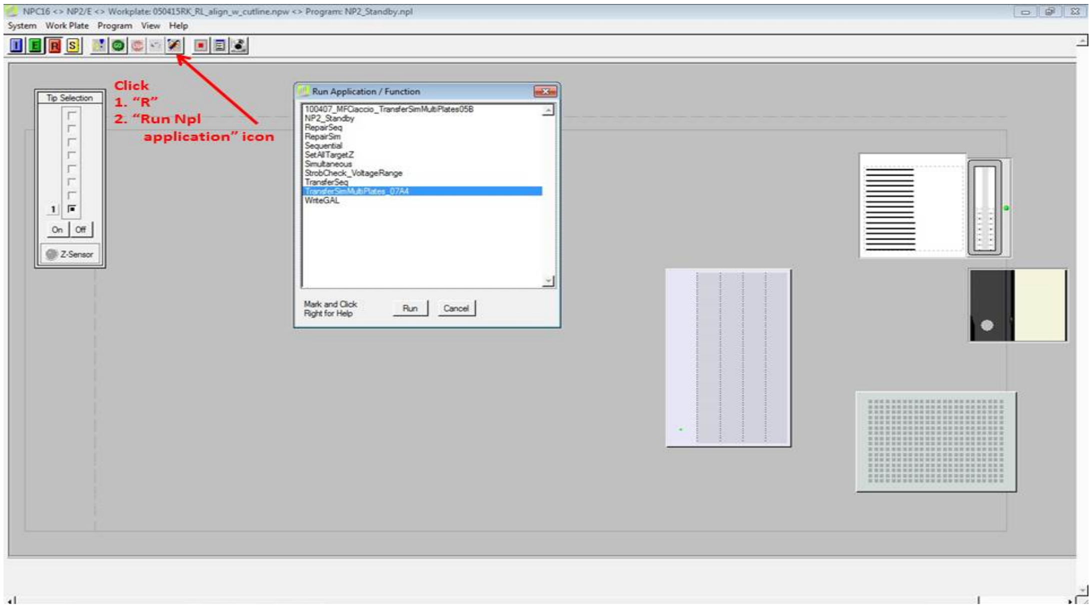

16) Check that the parameters in “Settings” window (see Fig. 6, below) are set to the following and then click “OK.”:

i) “Stroboscope check before spotting” checked.
ii) “passes” 4.
iii) “Stroboscope check after spotting” checked.
iv) “passes” 1.
v) “delay time” 1.0 sec
vi) “Dry before sample uptake” checked
vii) “Distance to target (spot) (mm)” 1.5.
viii) “Distance to target (move) (mm)” 1.5
ix) “Extra sample volume” 1.0
x) “Aspiration flow [uL/s]” 1.0
xi) “Wash time [s]” 9.

Fig. 6:"Settings Window"

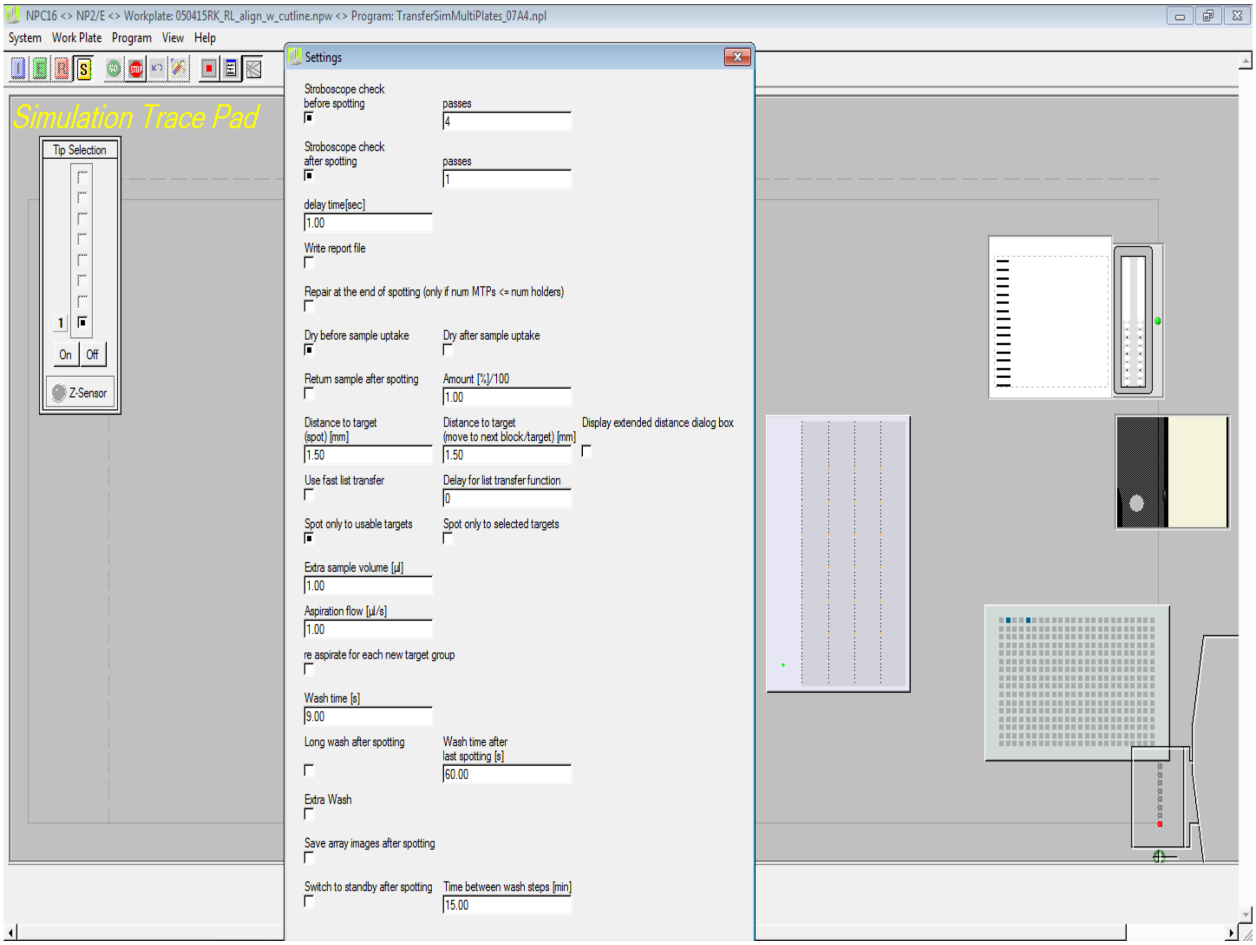

17) After clicking "OK" in the "Settings" window above, the following windows pop up:

a) Workplate objects – click "OK"

b) Open File – under "Look in" choose "RK transfer files", choose appropriate file based on number of samples and plate configuration and click "Select"

c) How to handle missing TG/TA/TI-com …" – all 4 options should be selected

d) Plate exchange type 16x24_45 – click "OK"

e) MTP content type 16x24_45-Plate 1 – click "No"

18) Printing of the gel will start using the transfer file chosen as the "Washing" window pops up.

a) Our transfer files include a line (“S9 TG[slides] BL1,2,3,4,25,26,27,28 15,1-15”) to allow bromophenol blue to be printed for measuring the distance samples travel during electrophoresis. (See SOP-A 13.0 “Gel Electrophoresis,” step 2k).

19) Printing can take several hours. Intermittently check (every ~30 min) that the printing is proceeding appropriately and that the chamber remains humidified for the duration of the print. Particularly long prints may require refilling the water tank used for tip washing.

20) After the printing is finished, leave the gel on the deck for ten minutes. This is to increase droplet adsorption.

21) Printed gel is ready for electrophoresis (see SOP-A 13.0 “Gel Electrophoresis")

22) QA/QC2. Following printing and between print runs, the NP2 standby program should be engaged to keep tips irrigated and hydrated until the next printing activity. Both the humidifier and arrayer water reservoir should be checked at least once a week, for adequate volumes of fluid. Turn off humidifier but keep computer, arrayer, and diluter on between prints.

Metadata

Record lot # for the following:

The specific lot #'s used in our experiments (as of July 2015) are:

1. Glycerol (Sigma #G5516) Lot#SHBG0744V

2. 10% SDS (Fisher #BP2436) Lot#121099

3. Sodium Bisulfate (Fluka #71656) Lot#BCBD7791V

4. DTT 1M (Fisher #AC42638) Lot#A0345819

5. Licor ladder (Licor #928-70000) Lot#C50311-06

Quality Assurance/Quality Control

QA/QC 1. If drop alignment is off then software will discard that particular drop. At the end of the print the software allows for running an "error" program to replace discarded drops. However, since the entire volume taken up by the tip for a drop is discarded after each cycle, there is a risk of not having enough sample in a well to replace the discarded drop.

QA/QC 2. Keeping tip irrigated and hydrated maximizes it's accuracy in a print.

Icahn School of Medicine at Mount Sinai LINCS Center for Drug Toxicity Signatures

Standard Operating Procedure:
Gel Electrophoresis for Microwestern Array

<u>DToxS SOP Index:</u> SOP A 13.0
<u>Last Revision:</u> 3/15/2017
<u>Written By:</u> Mark Ciaccio and Rick Koch

<u>Approvals (Date):</u> Joseph Goldfarb (DATE)
Marc Birtwistle (3/15/2017)
Eric Sobie (DATE)
Ravi Iyengar (DATE)

Quality Assurance/Control (QA/QC) steps are indicated with green highlight.

Metadata recording is highlighted with yellow highlight and superscript indices

1) Prepare reagents
   a) Rehydration buffer
      i) Combine 20 mL 5x Gel Buffer (SOP A 9.0, step *2a*), 20 mL glycerol (Sigma #G5516[1]) and 57 mL MilliQ water (Millipore Advantage A10 system 18.2 MΩ) in a 125 mL Erlenmeyer flask (Fisher, #FB-500-125).
      ii) Cover with parafilm (Fisher, #S37440) and mix vigorously by hand, shaking to ensure glycerol is evenly blended.
      iii) Add 1 mL 10% SDS (Fisher #BP 2436[2]) and gently mix by swirling.
      iv) Add 1 mL 1M NaBisulfate (6.00 g NaBisulfate (Fluka, #71656[3]) in a final volume of 50 mL of MilliQ water).
      v) Add 1 mL 1M DTT (Fisher, #AC42638[4]).
   b) Electrode buffer
      i) Combine 20 mL 5x Gel Buffer (SOP A 9.0, step *2a*), 29.5 mL of MilliQ water and 0.5 mL 10% SDS. Keep at 4°C. Prepare the day of electrophoresis.
2) Electrophoresis of printed gel
   a) Pre-chill the electrophoretic box (Gel Company #FC-EDCProf-2836) to 10°C with the Huber Minichiller (Huber, #3006.0029.99).
   b) While wearing gloves lift gel from the Nanoplotter deck (see SOP A 11.0, step 21) avoiding the printed area.
   c) Remove bottom plastic sheet from the printed gel by slowly peeling gel off sheet keeping the gel from curling in on itself.
   d) Place gel sample-side-up gently into 100 mL of Rehydration buffer in a glass tray (Daigger, #EF25365B) for 5 minutes.
      i) Gently submerge the entire gel with the sample side up.

ii) Wearing gloves, gently remove gel from rehydration buffer and vertically dab the gel bottom edge (along the D row of a gel used for a 24 well plate) on a paper towel for up to 5 seconds to remove excess buffer.
iii) Avoid pools of rehydration buffer on top of gel. If visible fluid is present leave gel on the electrophoresis box for 5 – 10 minutes to allow rehydration buffer to absorb into gel.
iv) QA/QC1 For prints that take 5 hours or longer (see SOP#A-11 0, Printing gel) the hydration time in step 2)d) should be increased to 12 minutes to avoid sample not migrating (sometimes referred to as "clumping" post-blotting).

e) Place the hydrated gel on the bed of the electrophoretic box with the sample side on top. Orient the gel so the proteins migrate to the red (positive) electrode.

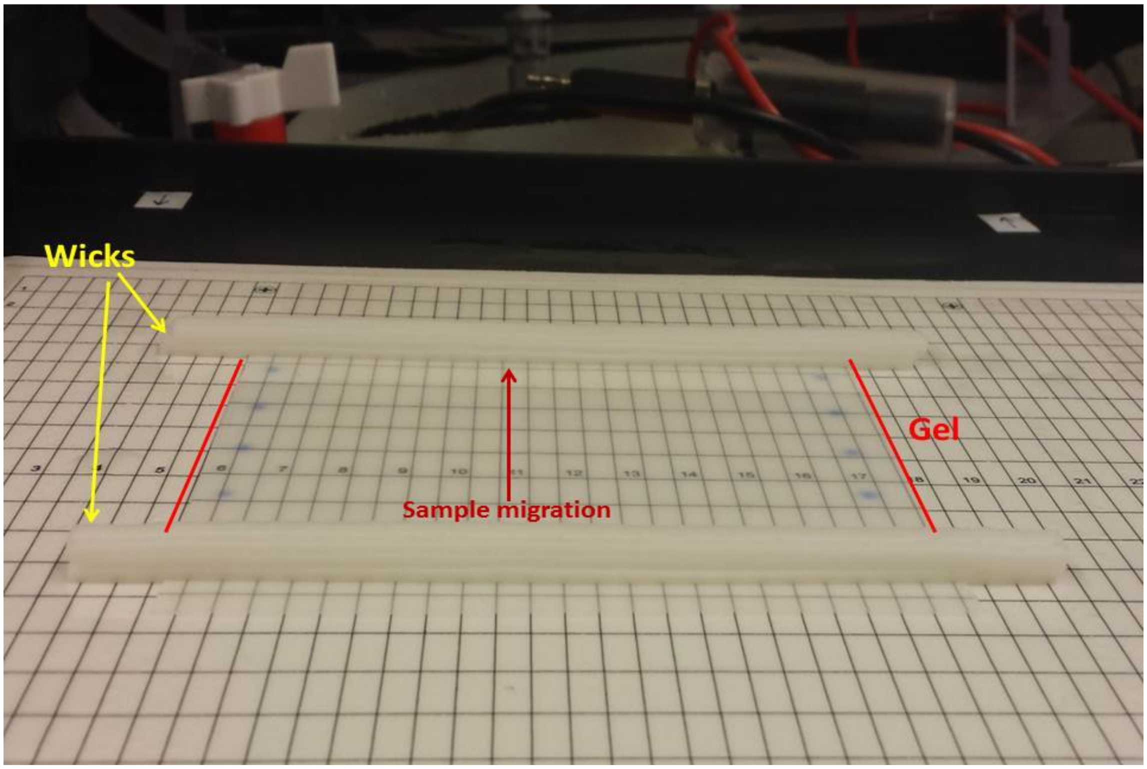

f) Cut 3 filter wicks (Fisher, #14056, Serva wicks, 300 x 6 x 1 mm) in half to give 6 150 mm long wicks and soak all in electrode buffer for several seconds.
g) Stack 3 wicks on top of one another and place on the top of the gel (above spots) perpendicular to the eventual voltage. Repeat for the bottom of the gel.
   i) Wicks should cover the length of the gel.
h) Run a gloved finger gently over the length of the wicks to make sure thorough contact is made with the gel.

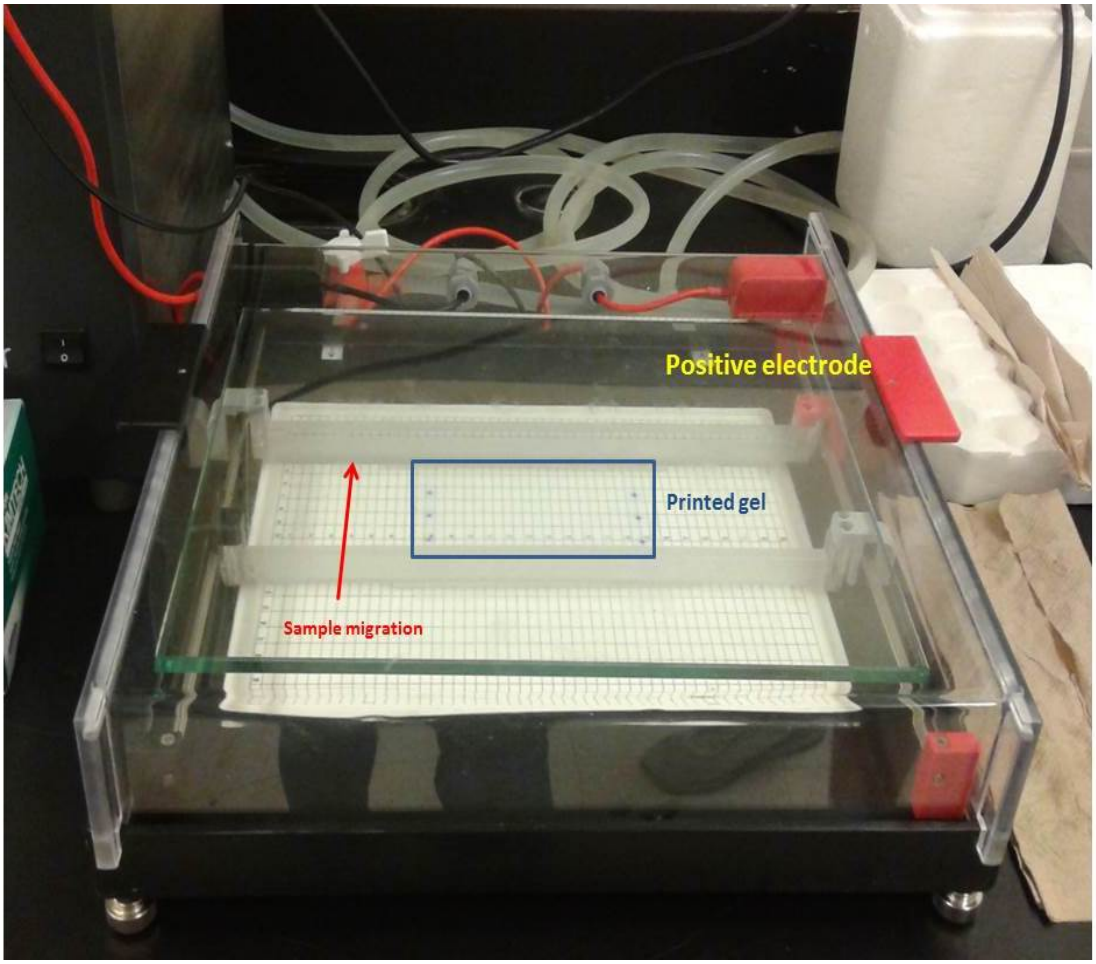

i) Place electrodes onto wicks and secure them with the heavy glass plate. Close the cover of the electrophoresis box.

j) Run the power supply at 60 V, constant voltage, for 5 min.

k) Turn the voltage up to 300 V (still at constant voltage). Stop the run when the bromophenol blue has run about 14 mm, approximately 30 minutes. (See SOP 11.0, Printing of Samples, step 18b).

i) Note: The time at 300V refers to a 24 well plate. This time will be shorter and should be independently determined for a 48 well or 96 well plate. Time will depend on the particulars of your equipment and setup so should be independently optimized. Particulars include the size of the wells (48 well size is 9mm x 18 mm vs the 18 mm x 18 mm for a 24 well plate) and the size of the proteins measured.

Metadata

Record lot # for the following:

The specific lot #'s used in our experiments (as of October 2016) are:

1. Glycerol (Sigma #G5516) Lot # SHBH0231V

2. 10% SDS (Fisher #BP 2436) Lot #121099

3. NaBisulfate (Fluka, #71656) Lot #BCBD7791V

4. 1M DTT (Fisher, #AC42638) Lot #A0345819

Quality Assurance/Quality Control

QA/QC 1. If gel is not properly hydrated sample will remain in the spot originally printed, failing to cleanly migrate down the gel as shown in the figure below (i.e. "clumping").

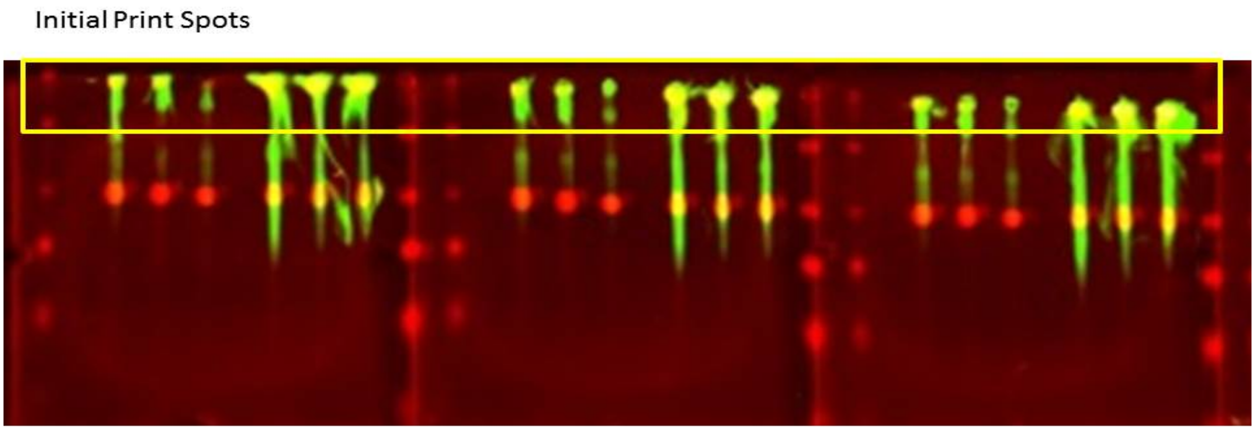

Icahn School of Medicine at Mount Sinai LINCS Center for Drug Toxicity Signatures

Standard Operating Procedure:
Wet Transfer to Nitrocellulose for Microwestern Array

DToxS SOP Index: SOP A 14.0
Last Revision: 03/15/2017
Written By: Mark Ciaccio and Rick Koch

Approvals (Date): Joseph Goldfarb (5/11/2017)
Marc Birtwistle (3/15/2017)
Ravi Iyengar (4/3/2017)

Quality Assurance/Control (QA/QC) steps are indicated with green highlight.

Metadata recording is highlighted with yellow highlight and superscript indices.

1) Prepare transfer buffer
   a. 10X Tris/Glycine
      i. Dissolve 30.2 g Tris (BioRad, #161-0719[1]) and 150.1g Glycine (BioRad, #161-0718[2]) in a final volume of 1 L MilliQ water (Millipore Advantage A10 system 18.2 MΩ). This can be kept at room temperature for several months (at least).
      ii. Measure pH; should be ~8.5. If less than 8.2 or greater than 8.8 start over with fresh Tris and Glycine.
   b. To make transfer buffer combine 300 mL methanol (VWR, # BDH1135-4LP[3]), 150 mL 10X Tris/Glycine and bring up to a final volume of 1.5 L with MilliQ water. Keep at 4°C.
2) Remove one “filter paper/Nitrocellulose (NC)/filter paper” sandwich (BioRad, #162-0233[4]) with gloves from box. Leave NC on top of one of the pieces of filter paper on clean bench surface. Immerse the other filter paper piece in 25 mL of transfer buffer.. Allow excess buffer to drain off and place filter paper on lab bench that has been cleaned with 70% ethanol.
3) Place a printed gel that has been subjected to electrophoresis (see Gel Electrophoresis SOP A 13.0) onto the damp filter paper, samples up, so that the sample region overlays filter paper as shown in Figure 1 below:

Figure 1. Printed gel overlaying blot

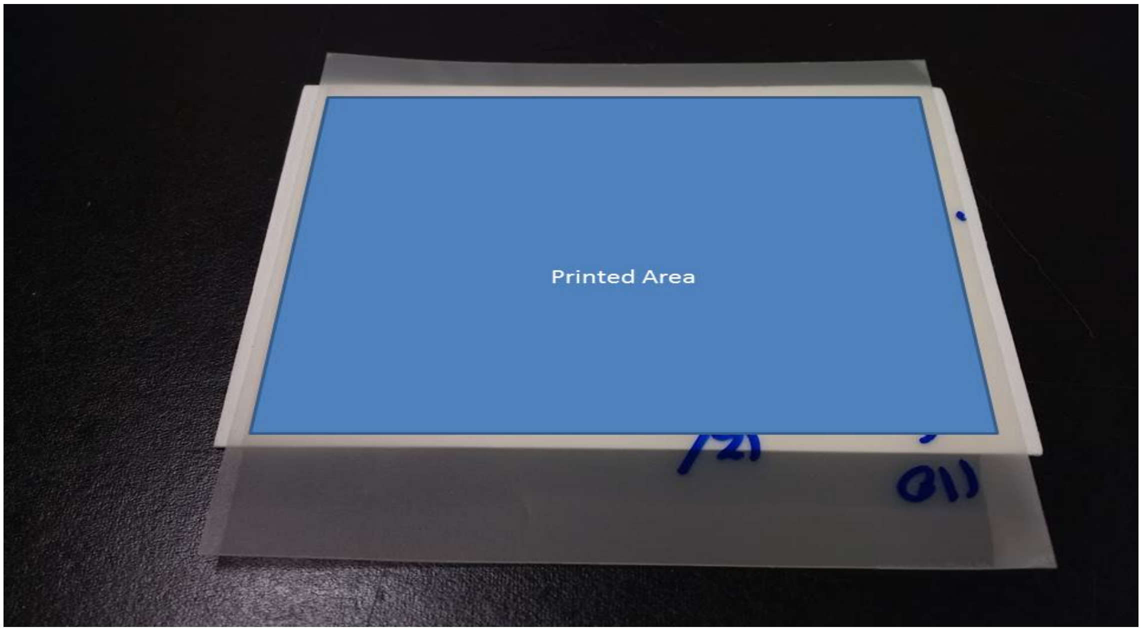

a. If an air bubble forms between the filter paper and gel, lift the gel and place back down so that gel lies evenly on the filter paper.

4) Wet the NC sheet of the sandwich in the same 25 mL transfer buffer, drain excess buffer and place onto gel. Wear gloves and take care not to fold or bend the NC.
5) Carefully roll out any bubbles (BioRad #1704070, Criterion blotter kit; roller is part of package) without moving the NC. This step is extremely important. Moving the NC after placing it on the gel may create a double or smeared image. Precise transfer is more critical than for a normal Western blot because much of the protein is on the surface of the gel after electrophoresis and will transfer instantaneously when touching the nitrocellulose.
6) Immerse second filter paper from the sandwich into the same transfer buffer, drain excess as before, and place on top of the NC. Carefully roll out any bubbles as in Step 5.
7) Place filter/gel/NC/filter sandwich between 2 foam pads and then into blotter gel holder cassette with NC closer to the red side (BioRad #1704070, Criterion blotter w/plate electrode kit which includes foam pads, ice block and gel holder cassettes).
8) Add ice block to Criterion Blotter tank, slide cassette into tank with red side of cassette facing red side of tank, and fill with transfer buffer; about 1.5 L is needed. Add stir bar.
9) Transfer sample from gel to nitrocellulose overnight with power supply set at 150 mA at 4°C while stirring (we place apparatus in the cold room).

Metadata

Record lot # for the following:

The specific control and lot #'s used in our experiments (as of July 20152016) are:

1. Tris (BioRad, #161-0719) Control # 210003844

2. Glycine (BioRad, #161-0718) Control #210012087

3. Methanol (VWR, # BDH1135-4LP) Lot # 081916E

4. (BioRad, #162-0233) Control #1201323

Icahn School of Medicine at Mount Sinai LINCS Center for Drug Toxicity Signatures

Standard Operating Procedure:
Quantifying Microwestern Array Images

DToxS SOP Index: A 16.0
Last Revision: 01/03/2018
Written By: Rick Koch
Approvals (Date): Joseph Goldfarb (1/3/2018)
Marc Birtwistle (1/3/2018)
Ravi Iyengar (1/23/2018)

Quality Control (QC) steps are indicated with green highlight.

Metadata recording is highlighted with yellow highlight and superscript indices.

----

1) Export image from Li-Cor Odyssey scanner as a zip file and import into "Image Studio Lite Ver. 5.2," (free download from Li-Cor).[1]
    a. Import by clicking "iS" icon, highlight "Import," click "Image Studio Zip and select exported image. (See Figure 4.a. below.)

Fig. 1 Jpg Image of Zip file from Odyssey scanner

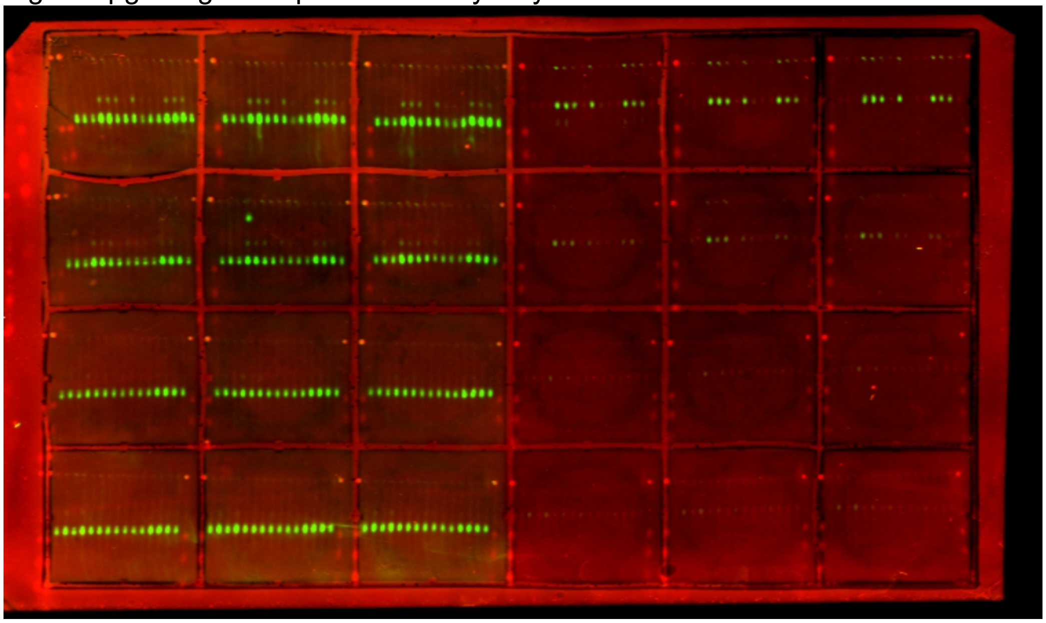

2) Be sure image is oriented to match the right/left and up/down coordinates of the original sample print. See SOP# A-11, "Printing of Samples for Microwestern Array." This is aided using an "Antibody Plate Map" which shows which Ab(s) are used in which wells, at what dilution and with what color secondary. Example related to image above shown below:

Figure 2, Ab Plate Map for MWA run on 091516 as shown in Figure 1 and image below.

2016 0915 Ab Plate Map — Rabbit / Mouse

	1	2	3	4	5	6
A	Erk 1/2	Erk 1/2	Erk 1/2	Akt (pan)	Akt (pan)	Akt (pan)
	Phospho Erk	Phospho Erk	Phospho Erk	Phospho Akt	Phospho Akt	Phospho Akt
B	Erk 1/2	Erk 1/2	Erk 1/2	Akt (pan)	Akt (pan)	Akt (pan)
	Phospho Erk	Phospho Erk	Phospho Erk	Phospho Akt	Phospho Akt	Phospho Akt
C	Erk 1/2	Erk 1/2	Erk 1/2	Akt (pan)	Akt (pan)	Akt (pan)
	Phospho Erk	Phospho Erk	Phospho Erk	Phospho Akt	Phospho Akt	Phospho Akt
D	Erk 1/2	Erk 1/2	Erk 1/2	Akt (pan)	Akt (pan)	Akt (pan)
	Phospho Erk	Phospho Erk	Phospho Erk	Phospho Akt	Phospho Akt	Phospho Akt

Protein	KDa
Akt (pan)	60
Phospho Akt	60
Erk 1/2	42/44
Phospho Erk	42/44

Ab	Source:
Akt (pan)	CS #2920S(40D4)
Phospho Akt	CS #4060L(Ser473)(D9E)
Erk 1/2	CS #4697S (p44/42 MAPK)(3E1)
Phospho Erk	CS #4370 (p44/42MAPK)

Ab	Dilution
Akt (pan)	1/1K
Phospho Akt	1/2K
Erk 1/2	1/500
Phospho Erk	1/1K

Figure 3, blow up of Well D1 showing the 15 samples printed in that well.

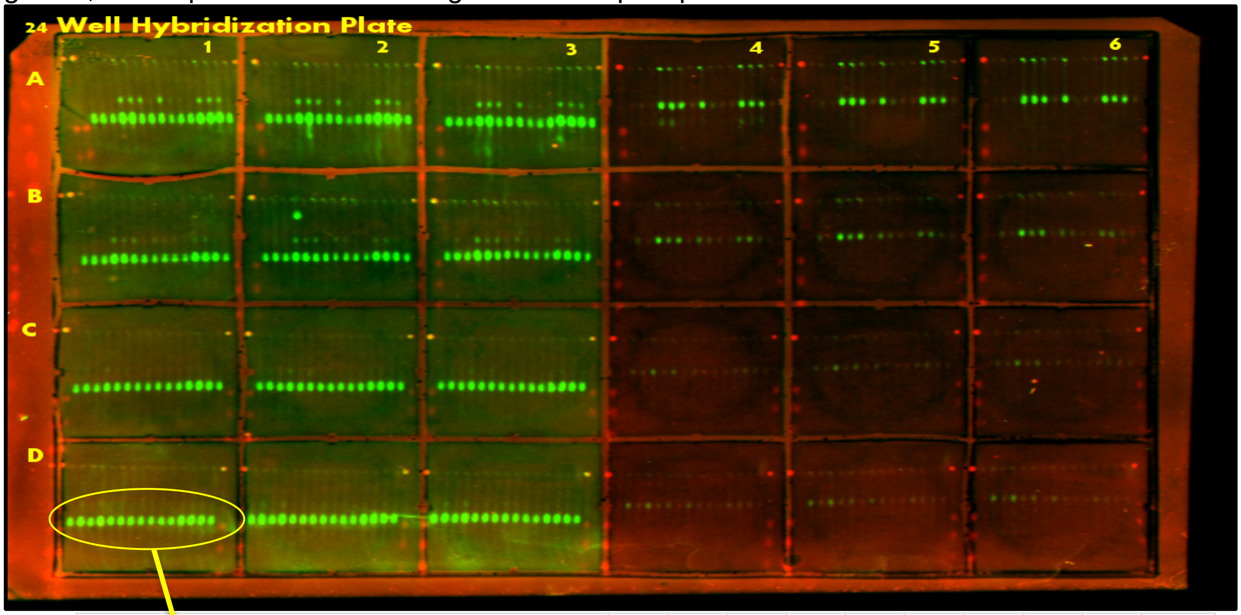

2016 091[illegible]5 MCF10A's: Samples per lane per well(s)

Wells:	Lanes:															
D1 - D6	17	16	15	14	13	12	11	10	9	8	7	6	5	4	3	2	1
Sample #	Ladder	15	14	13	12	11	10	9	8	7	6	5	4	3	2	1	Ladder

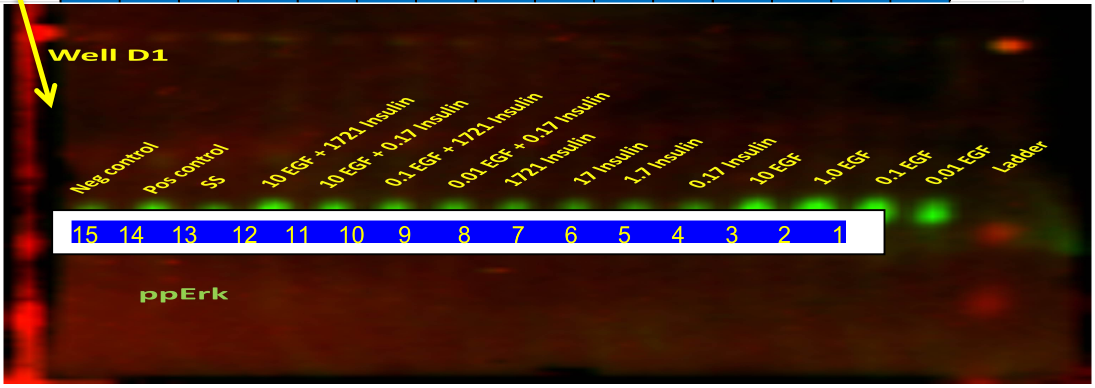

3) Analyze scan (refer to Figures 4a. and 4b below, “Image Studio Lite Analysis Example”).
   a. Click “Analysis” tab.
   b. To measure the 700 signal (“red,” typically anti-mouse secondary Ab in our experiments), deselect the 800 signal. To measure the 800 signal (“green,” anti-rabbit secondary Ab), deselect the 700 signal.
   c. Click “Add Rectangle.” Position mouse arrow over signal and click to get rectangle on signal.
   d. Adjust rectangle to measure signal strength.
      i. Click the “Select” button in the menu. (See Figure 4a below.)
      ii. Click “Profiles” tab along right side of Image Studio Lite window. (Window goes from Fig. 4a to Fig. 4b) Return cursor to the signal/rectangle and click to highlight it. When cursor becomes a crosshair the rectangle can be moved. By placing cursor in the corners, an arrow appears allowing the size of the rectangle to be adjusted.
      iii. Adjust the size of the rectangle so that the intensity distributions for the X and Y locations (see right panel in Fig. 4b below) have the correct cut-offs (where graphs intersect with the y and x baselines or as close to the baseline as possible; if signals are close to each other along the x – axis (left/right) then the intensity distribution may not intersect with the axis).
   e. See Figure 4c below. Background is determined by clicking the “Median . . .” symbol above “Background” panel. In popup window “Background,” choose “2” for Border Width and the button “Top/Bottom,” and then click “Save.” Background is subtracted automatically.
      i. Note: Top/Bottom is selected for background because this is the direction of the electrophoresis lane, which tends to be the predominant background contribution.
   f. Click “Shapes” tab below image window (bottom left corner of image window to right of “Images” tab). In “Signal” column the signal strength value will be displayed.(Fig 4c)
   g. Prepare a separate Excel spreadsheet and copy the values from the “Signal” column to the appropriately labelled cell of that Excel spreadsheet (see Figures 4b and 5 below). Each signal is measured and recorded in this way. Table represents phospho-ERK1/2 data from the first four lanes in Well D1 of Figures 1 and 2

Figure 4a, Image Studio Lite Analysis Example

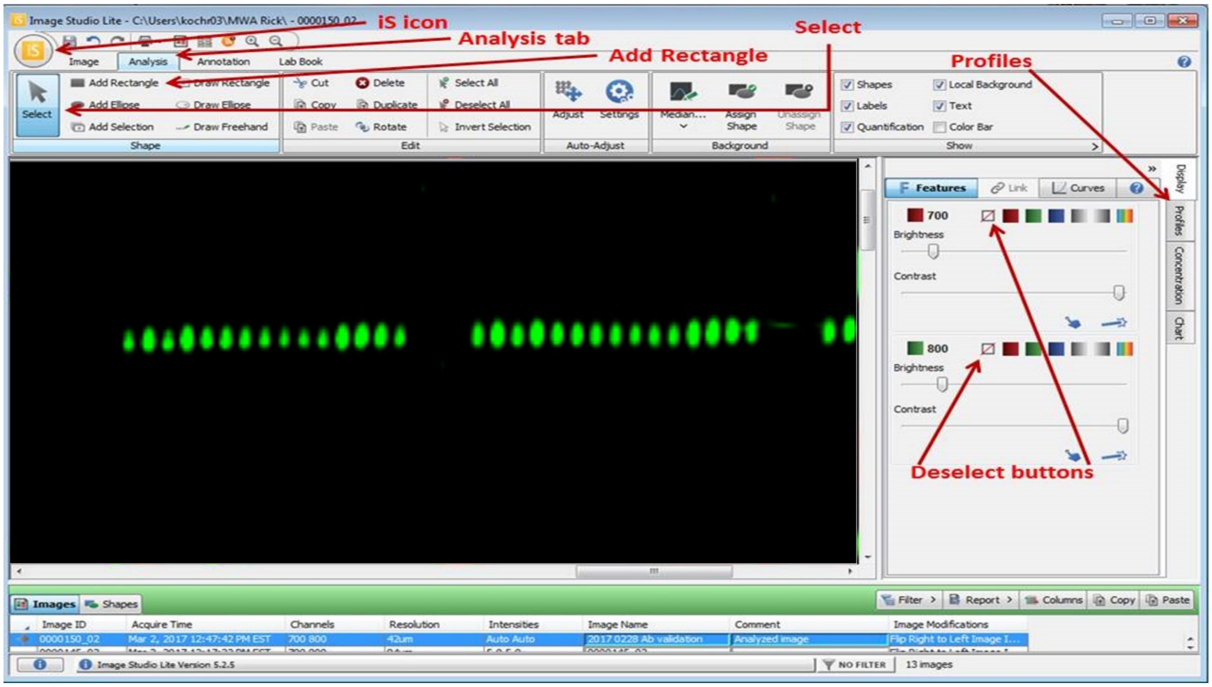

Figure 4b Image Studio Lite Analysis Example

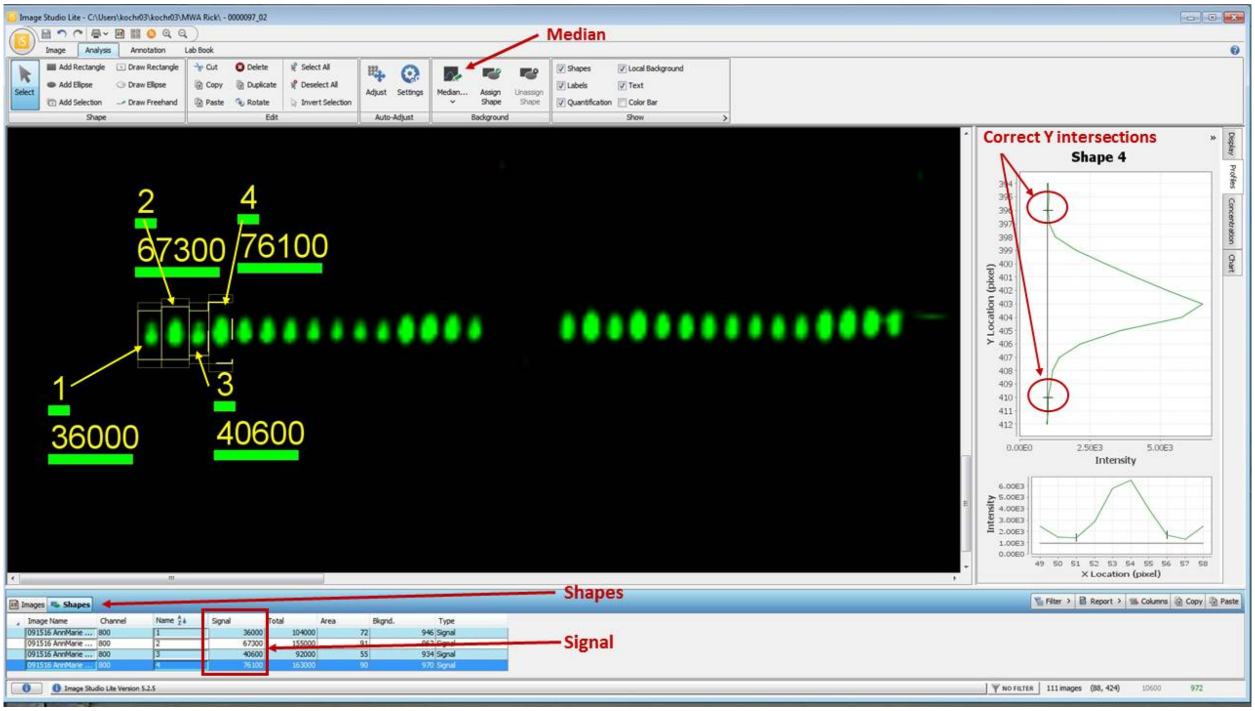

Figure 4c Image Studio Lite Analysis Example

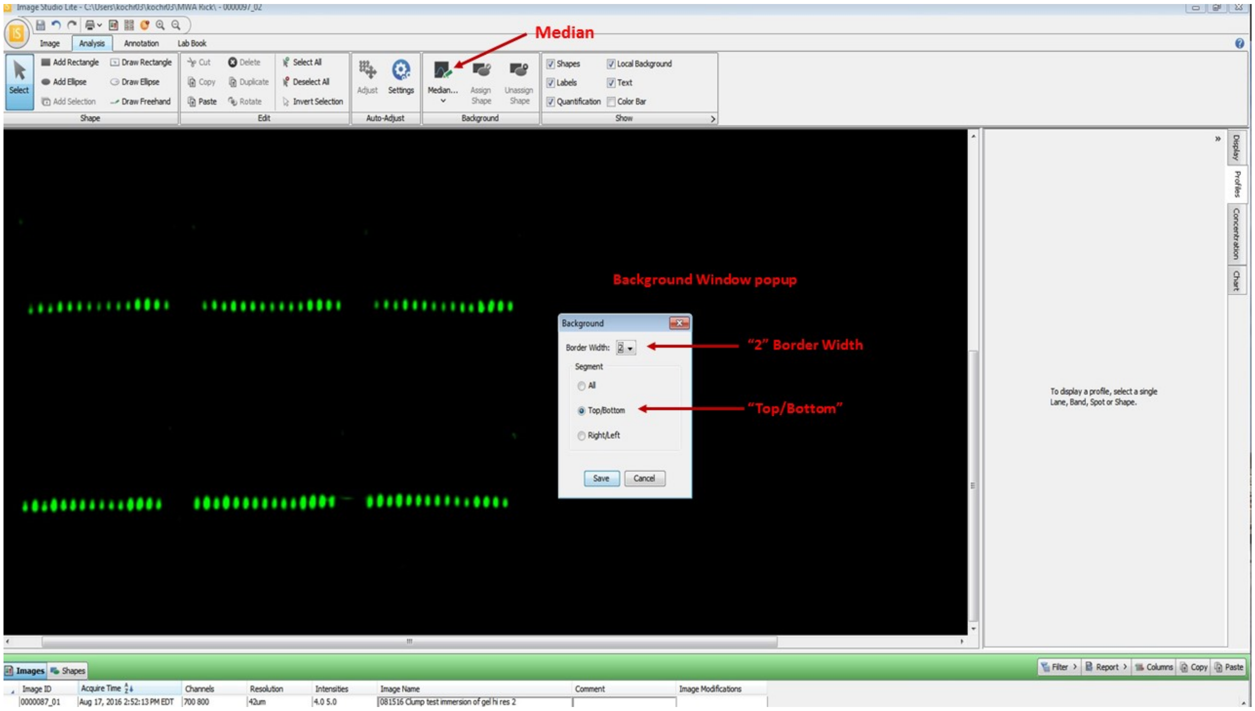

Figure 5 Example of Excel Spread sheet data (Entries in yellow represent spots 4,3,2,and 1 in Fig 4b.)

091516 MCF10A cells treated with EGF and Insulin at different times pts / Analysis using Image Studio						
	phospho ERK					
6 hours	D1	D2	D3	Average	S.D.	C.V.
EGF 0.01	28400	42500	28200	33033	8199	25%
0.1	54700	70900	48400	58000	11607	20%
1	66500	75500	55200	65733	10172	15%
10	67700	68700	55100	63833	7580	12%
Insulin 0.1	29400	33900	26000	29767	3963	13%
1	28600	36500	28100	31067	4712	15%
10	27600	33700	23100	28133	5320	19%
1721	32200	36200	35600	34667	2157	6%
E+I 0.01/0.1	38200	41000	40000	39733	1419	4%
0.01/1721	39000	49700	46400	45033	5479	12%
10/1.0	47800	54600	51300	51233	3400	7%
10/1721	76100	78300	76200	76867	1242	2%
SS	40600	44300	43200	42700	1900	4%
Pos control	67300	69100	63000	66467	3134	5%
Neg Control	36000	40900	41800	39567	3121	8%

Metadata

1. . “Image Studio Lite Ver. 5.2,” (free download from Li-Cor: https://www.licor.com/bio/products/software/image_studio_lite/download.html), (Licor).
